# The interaction between land‐use change and fire regimes, directly and indirectly, affects the urban avian assemblages of Darwin, Australia

**DOI:** 10.1002/ece3.10239

**Published:** 2023-07-03

**Authors:** Sarah Fischer, Andrew C. Edwards, Stephen T. Garnett, Timothy G. Whiteside, Patrice Weber

**Affiliations:** ^1^ Faculty of Science and Technology, Research Institute for the Environment and Livelihoods Charles Darwin University Darwin Northern Territory Australia; ^2^ Faculty of Science and Technology, EIE Environment Charles Darwin University Darwin Northern Territory Australia; ^3^ Environmental Research Institute of the Supervising Scientist Darwin Northern Territory Australia

**Keywords:** avian assemblages, fire, land‐use change, stressor interactions

## Abstract

The interaction between environmental stressors may be a greater threat to biota than any individual ecological threat on its own. Land‐use change and inappropriate fire regimes are known to pose great challenges to biodiversity conservation worldwide. Despite much research being conducted into their singular impacts on ecosystems, very few have investigated how their interaction may be affecting the biota of a region. We used data from surveys in 1998/2000 and 2019/2020 to compare the feeding guild assemblages of bird communities in different habitats within the greater Darwin region. By compiling two sets of spatial data, land‐use change, and fire history mapping, we were able to investigate their interaction and impact on the avian assemblages in the Darwin urban area. Using Generalized Linear Mixed Models (GLMM) we found that an increase in urbanization significantly affected fire occurrence across study sites. Furthermore, we found that the interaction between land‐use change and fire regimes had a significant effect on species that primarily feed on fruit. We conclude that while an increase in urbanization did not directly affect the avian assemblages, the impact of land‐use change on the fire regimes indirectly impacted urban bird community structures.

## Graphical abstract

Using spatial data and historical and recent bird surveys, we investigated the impact of land‐use change and fire on the Darwin bird assemblages. We determined that the interaction between land‐use change and fire regimes directly affected frugivores. Furthermore, we found that the effect of land‐use change on fire regimes indirectly impacted avian assemblages.

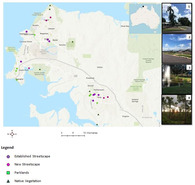

## 1 INTRODUCTION

Globally, overexploitation (the unsustainable harvesting of wild species), agriculture, and urban development are considered the major threats to biodiversity (Maxwell et al., 2016), with pollution, invasive species, climate change, and ecosystem modification (such as changes to hydrological and fire regimes) also of significant concern. Threats to Australian biodiversity follow these global trends but the greatest dangers are considered to be invasive species, ecosystem modification, particularly altered fire regimes, and agriculture (Kearney et al., 2019). The exceptionalism of Australia is corroborated by the most recent “Australia State of the Environment” report (Cresswell et al., 2021) which asserts that nearly 70% of Australia's threatened species are imperiled by changing fire regimes. Increases in the frequency and intensity are most frequently identified as threats (Franklin et al., 2005; Kelly et al., 2020; Wills et al., 2020) with many studies following single intense fires (Harley, 2021; Hyman et al., 2020; Ward et al., 2020). However, a reduction in both intensity and frequency can also affect faunal assemblages, usually as a result of changes in vegetation structure and composition (Perry et al., 2016; Regan et al., 2010).

A number of studies have examined the impacts of urbanization on faunal assemblages (see Champness et al., 2019; Major & Parsons, 2010; Sol et al., 2017, among others), often focusing on losses resulting from the initial land development through to investigating the re‐establishment of fauna, or the arrival of new species, as parks and gardens mature (Catterall et al., 1998; Lim & Sodhi, 2004).

Where studies have considered both fire and urban environments, they have largely followed major conflagrations that have consumed the urban fringes, possibly because fire management in such areas has concentrated on fire suppression.

From the 162 papers describing 274 cases reviewed by Driscoll et al. (2021), there were only 12 cases in which the interactions between fire and habitat loss or fragmentation were comprehensively reported, even with a very broad definition of ‘fragmentation’, and most of the studies (58%) were simulations rather than empirical.

In this study, we fill this gap in the understanding of the interaction between fire and urbanization by investigating temporal changes in avian communities across a variety of habitats within the Greater Darwin region in northern Australia.

Darwin, the only capital city in Australia where urban fires are a regular annual occurrence, is located within the monsoonal tropics. The landscape is dominated by tropical savannas (discontinuous tree cover and continuous grass) in which fires occur frequently during the prolonged dry season from May to October, often very close to suburban housing areas (North Australia and Rangelands Fire Information, 2018). Many of these fires are prescribed, being undertaken by various land management bodies as part of a yearly landscape management approach (Evans et al., 2019; Russell‐Smith et al., 2020). However, rapid population growth and associated urbanization of the region are likely to mean that the timing, frequency, and intensity of fire differ from what would have occurred in the landscape before it was developed. Nevertheless, an earlier study by Fischer et al. (2021) investigating the impact of 20 years of land‐use change on Darwin bird assemblages generally, determined that increasing urbanization had no significant effect on guild assemblages; however, as with prior research, this study focused on land‐use change alone.

Here, we determine how local bird populations are affected by the combined effects of urbanization and altered fire regimes, by investigating (1) whether land‐use change is affecting the fire regime (likelihood of fire occurrence, area burnt, seasonality of fire); (2) whether there is an interaction between changing land use and fire regime that results in a combined effect; and (3) how any interactions may be affecting feeding guild structures within avian assemblages.

## 2 MATERIALS AND METHODS

### 2.1 Study area

Darwin is located on the northern coast of Australia (12.4634°S, 130.8456°E). The climate is that of the monsoonal tropics comprising distinct dry and wet seasons (May–Sept and Nov–Apr, respectively) with transitional periods in between (Bureau of Meteorology, 2022). Approximately 1700 mm of rain seasonally is recorded on average, and maximum mean daytime temperatures range from the low to high thirties (°C). The estimated resident population of Darwin as of June 2021 was 148,800, an increase in around 40% over the past 20 years (Australian Bureau of Statistics, 2022), during which time the urbanized area increased from 114 to 151 km^2^ (Fischer et al., 2021).

Highly flammable tropical savanna woodlands are the dominant habitat in the greater Darwin region with other habitats including monsoon forests, both wet (alongside waterbodies) and dry, large areas of mangrove vegetation and tracts of green space containing native and introduced tropical plant species supporting an array of fauna (Brock & Dunlop, 1988; McCrie & Noske, 2015).

### 2.2 Historical and recent bird survey data

**FIGURE 1:**
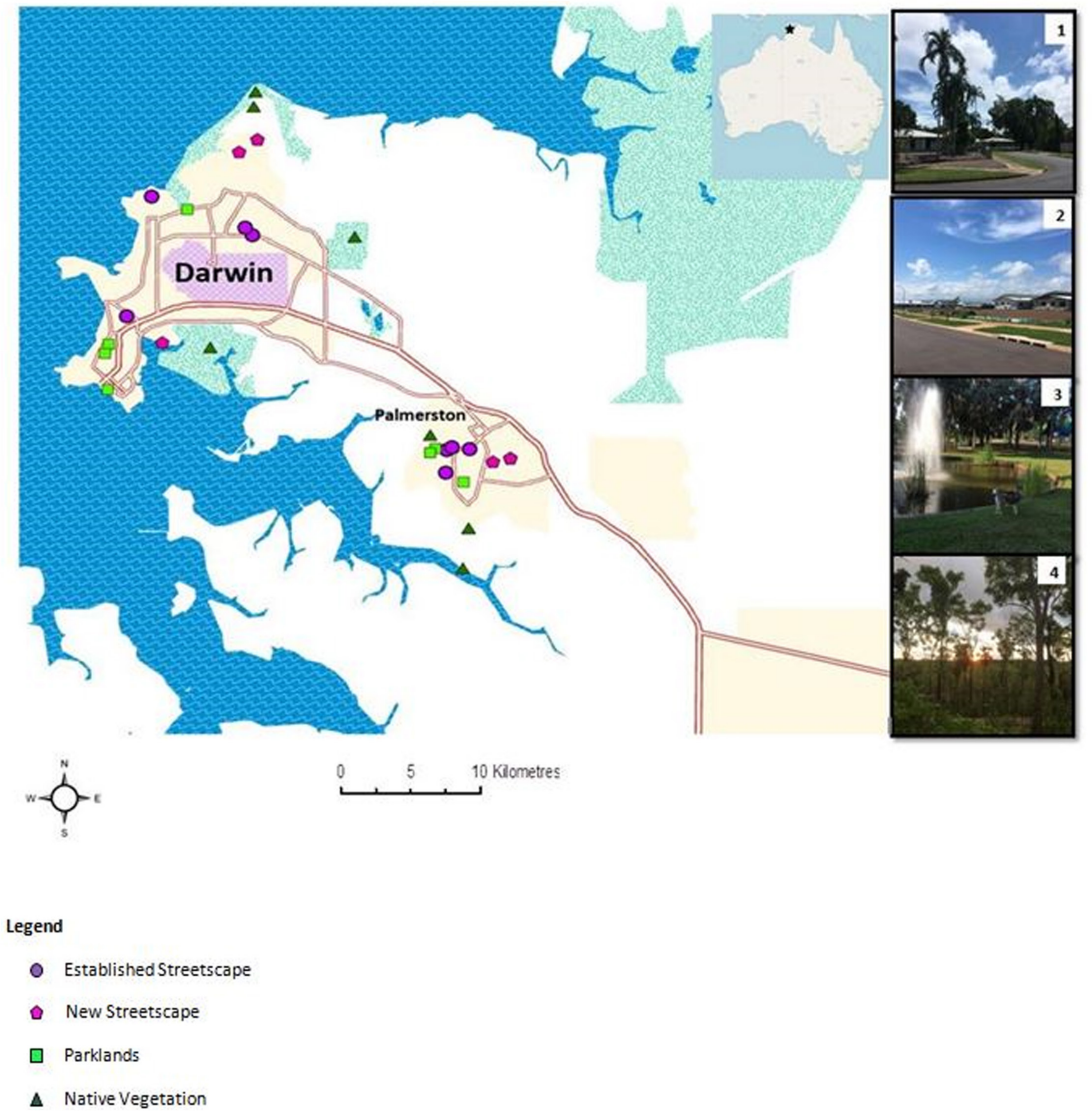
Location map of study sites within the greater Darwin region and examples of habitat types (photographs S. Fischer).

Both conventional and novel point‐count and line‐transect surveying techniques were employed when conducting bird surveys in 32 sites during 2019 and 2020. Conventional surveying was that of stationary observer recording (point‐counts) and walking pre‐determined transect lines. Novel surveying techniques involved bioacoustic recording of birds, both stationary (point‐counts) and moving (line‐transect) via the use of a drone. Four distinct habitat types, eight sites of each, were surveyed (Figure 1):

1. Established Streetscapes: urban or suburban areas established before the year 2000 where no further significant development is anticipated. Greenspace and gardens are made up of mature vegetation, both native and exotic
2. New Streetscapes: urban or suburban areas where development did not begin until 2001 or later and may be continuing. Relatively recent clearing of native vegetation, few mature plants in gardens;
3. Parklands: recreational green space excluding those areas used for sporting activities;
4. Native Vegetation: areas comprising tropical savannas and remnant monsoon vine forests; minimal or no development is planned for these areas.

Species detectability was examined overall and within the different habitat types and is described fully in Fischer et al. (2023).

The species composition of recent survey data was compared to bird survey data extracted from the BirdLife Australia “Atlas of Australian Birds” (henceforth known as the Bird Atlas; BirdLife Australia, 2022) database for the years 1999 and 2000 for the locations as described for the land‐use mapping. As the 2019/2020 surveys were focused on terrestrial habitats surveyed during the day, Bird Atlas records were filtered to exclude all aquatic and nocturnal species and species considered “vagrant.” At the time of writing no introduced bird had become established in Darwin. Overall, 180 individual species were identified. The majority of these were in the order Passeriformes (42%) with the next most populous order being that of Charadriiformes (17%; Table A1).

### 2.3 Spatial data

Two sets of spatial data were compiled for the study sites: urban/greenspace land‐use change and fire history mapping. Land‐use mapping for a 1 km buffer centered around the coordinates of the 32 study sites was derived from a mixture of Landsat 4–5 Thematic Mapper (TM) and Landsat 7 Enhanced Thematic Mapper Plus (ETM+) imagery for the years 1998 to 2012, and Landsat 8 Operational Land Imager (OLI) for the years 2013 to 2018 (ground sample distance [GSD] 30 m); fire mapping employed the same imagery as well as Sentinel‐2 imagery for the years 2016–2018 (GSD 20 m).

#### 2.3.1 Urban and greenspace land‐use change

Because land‐use change can occur slowly and be difficult to see at smaller scales, differences were measured across four time periods (from 1998 to 2003, 2003 to 2007, 2007 to 2013, and 2013 to 2018) and overall (from 1998 to 2018). The Landsat imagery covering the study area was sourced via the United States Geological Survey's Global Visualization Viewer (GloVis) and processed using the methods as described in Fischer et al. (2021), in ArcGIS version 10.4.1 (Environmental Systems Research Institute [ESRI] 1999–2015) and SAGA GIS version 7.9.0 (Conrad et al., 2015). Depending on the availability of coverage and image quality, images were selected from April or May when the wet season is ending, cloud cover is minimal and photosynthetically active growth is still at its peak with minimal fire scarring. Using geographic object‐based image analysis (GEOBIA), segmented pixels (pixels with similar spectral characteristics) were assigned to the following land types: suburban, periurban, buildings, roads, grass, woodland, forest, mangrove, sea, sand, water, and bare earth; the area (m^2^) of each category within each study site zone was calculated in ArcGIS. The categories of suburban, periurban, buildings and roads were combined in the category “urbanization” and grass, woodland, and forest combined for “greenspace.” The differences in urbanization and greenspace were measured by comparing relevant land class area data from the maps between the various periods. Accuracy assessments of the classified images were undertaken as per Fischer et al. (2021), using standard methods (Congalton, 1991). Random accuracy assessment points were generated, and an error matrix was constructed to assess User (supervised classification by the map maker) and Producer (unsupervised classification by the SAGA GIS program) accuracy when assigning classification. Aerial photographs, satellite imagery and Google Earth Pro version 7.3.4.8642 (64‐bit) were used to provide reference data when checking map classification accuracy.

Land class areas between the time periods were calculated in m^2^ and expressed as a proportion of the 1 km buffer surrounding the study site coordinates.

#### 2.3.2 Fire history of study sites

A fire history of the study sites indicating the intra‐seasonality of fires during the fire season (early, mid, and late dry season), along with the proportion of the study site burnt, was determined by calculating the Mid‐Infrared Burn Index (MIRBI) and the differenced Mid‐Infrared Burn Index (dMIRBI) between satellite image dates (Table 1).

**TABLE 1:** Spectral indices and band designations used in fire regime analysis.

Spectral index	Formula
MIRBI Mid‐infrared Burn Index	*MIRBI =* ((*SWIR1 × 10*) *−* (*SWIR2 × 9.8*) *+ 1*)
dMIRBI differenced Mid‐infrared Burn Index	*dMIRBI = MIRBI*_*TIME2*_ *− MIRBI*_*TIME1*_
**Imagery Source**	**Bands used**
Landsat 4–5 TM & Landsat 7 ETM+	Band 7 (SWIR1) Band 5 (SWIR2)
Landsat 8 OLI	Band 7 (SWIR1) Band 6 (SWIR2)
Sentinel‐2	Band 12 (SWIR1) Band 11 (SWIR2)

MIRBI was chosen as it is a spectral index that incorporates short‐wave infrared reflectance (SWIR) wavelengths. Compared with those indices that use only visible or near‐infrared (NIR) reflectance, such as the Normalized Difference Vegetation Index (NDVI) and the Enhanced Vegetation Index (EVI; Smith et al., 2007), SWIR is better at detecting fire scars when the water content of vegetation is high, such as that found in the late wet/early dry season period of the Darwin region. Furthermore, MIRBI is more sensitive to the spectral changes caused by fire in savanna landscapes yet has low sensitivity to the intrinsic variability of ecological systems (Trigg & Flasse, 2001). As with land‐use change, burnt areas were measured in m^2^ and expressed as a proportion of the 1 km buffer around the study site coordinates.

The remote sensing platform Google Earth Engine (GEE) was used to access and process images from its extensive publicly available catalog of Landsat and Sentinel 2 imagery. Using GEE code provided (North Australia and Rangelands Fire Information, 2018), images were filtered by the region of interest and level of cloud cover for each year. Every cloud‐free, or minimal cloud cover, image was chosen and the MIRBI calculated for each. MIRBI images were added to the map display and a dMIRBI image was created by subtracting MIRBI values between two sequential image dates. The resultant dMIRBI image was then reclassified to highlight burnt areas and converted to a vector layer. MIRBI images and the reclassified dMIRBI vector polygons outlining the burnt area mapping were then imported into ArcMap. The vector polygons were intersected with polygons delineating the study site areas, and the area burnt at the site was calculated.

Fire maps were validated following Sparks et al. (2015) where reference data were created by way of visual interpretation of false color composite images and were analyzed using RGB combinations as shown in Table 2 (USGS, 2020).

**TABLE 2:** RGB band combinations of Landsat and Sentinel‐2 imagery used for fire map validation.

Imagery source	Combination	Function
Landsat 4/5/7 (1998–2012)	Band 7	SWIR—Hydrothermally altered rocks associated with mineral deposits
	Band 4	Near‐Infrared—Emphasizes biomass content and shorelines
	Band 3	Red—Discriminates vegetation slopes
Landsat 8 and Sentinel‐2 (2013–2021)	Band 7	SWIR2—Improved moisture content of soil and vegetation; penetrates thin clouds
	Band 5	Near‐Infrared—Emphasizes biomass content and shorelines
	Band 4	Red—Discriminates vegetation slopes

One image per year was chosen for validation. Using randomly generated accuracy assessment points, pixels were visually assigned simply as either burnt or unburnt and then compared to the results of the relevant dMIRBI. Determination of the pixel classification was aided by NAFI MODIS‐derived burnt area mapping, aerial photography, satellite imagery, and Google Earth Pro version 7.3.4.8642 (64‐bit).

### 2.4 Statistical analysis

Land‐use change was recorded as either an increase, decrease, or no change in the amount of land categorized as urbanized (suburban, periurban, buildings, and roads) or greenspace (grass, woodland, forest); changes were recorded for each time period (1998–2003, 2003–2007, 2007–2013, and 2013–2018) and overall (1998–2018). To determine the effect of changing land use on fire regimes, the total number of years that fire occurred at a site was recorded for each land‐use change time period (“fire occurrence”) and expressed as a proportion; the average proportion of the total land area of the site burned (‘mean % burnt’) for each land‐use change time period was also calculated. Fire data were then separated into the early, mid, or late dry season, to encompass the annual fire season, and the fire occurrence and the area burnt were again calculated with regard to the seasonality of fire. As with land‐use changes, changes to the fire occurrence and mean % burnt were noted as an increase, decrease or no change. Changes in the incidence and timing of fire were compared using a Generalized Linear Mixed Model (GLMM) where we included urban and greenspace change (increase, decrease, no change), and interactions between urban and greenspace change and habitat type (Established Streetscape, New Streetscape, Parkland, and Native Vegetation), as fixed effects.

Bird species were categorized by their primary food source, that is, the singular food source predominating the diet, similar to Fischer et al. (2021). Primary food source determination, fruit, invertebrates, nectar, omnivore, raptor, seed, vegetation, or vertebrate, was based on information from Australian Bird Data Version 1.0 (Garnett et al., 2015), BirdLife Australia (2022) and the Atlas of Living Australia (2021). The feeding guild assemblages were then compared between the two survey periods. To allow for meaningful comparison, sites that had species records of five or fewer were omitted from the analysis. The change in feeding guild member proportions between the 1999/2000 and 2019/2020 surveys was used to investigate the effect of land‐use change and fire regimes on bird communities within the study sites. Guild member proportions were used instead of species population numbers as the 1999/2000 data extracted from the Bird Atlas was gathered from scores of bird surveys undertaken by many observers, whereas data from 2019/2000 was that of a single observer.

We again employed a GLMM to compare the change in guild member proportions by primary food source. As with the fire regime analysis, urban change and greenspace change were fixed effects but we also included the overall change in the fire occurrence and the mean % burnt (increase, decrease, no change); interactions between land‐use change and fire regimes and land‐use change and habitat type were again examined.

DHARMa residual diagnostics was used to assess the suitability of the models; additionally, residuals were checked for autocorrelation and found to be independent (*p* > .05).

Data were analyzed using the “lme4 R Stats Package,” version 4.0.1 in RStudio (R version 4.0.1 [2020‐06‐06]; RStudio Team, 2020).

## 3 RESULTS

### 3.1 Spatial data

#### 3.1.1 Accuracy of spatial data

The accuracy of final maps produced using GEOBIA land‐use classification derived from satellite imagery for urban and greenspace change ranged from 74.5% to 82.5%; burnt area mapping using dMIRBI classification ranged from 93.0% to 97.0% (Table 3).

**TABLE 3:** Average accuracy (%) of spatial maps created to assess land use and fire history.

Time period	Land‐use classifications	Burnt area mapping
1998–2003	81.3	93.0
2003–2007	74.5	94.6
2007–2013	75.0	95.6
2013–2018	82.5	97.0
All dates	78.4	94.9

#### 3.1.2 Urban and greenspace land‐use change

Of the 32 survey sites, 24 showed an overall increase in urban space from 1998 to 2018 with the greatest percentage increase found in those areas categorized as New Streetscapes (up to 58%). Greenspace increased in 11 sites, the highest being in an area classed as Native Vegetation (26%), but some considerable increases were recorded in two of the New Streetscape sites (16% and 20%). The greatest decrease in greenspace was also found in New Streetscape sites (42%). No change to urban land use was found in five sites, and one site showed no change to the amount of greenspace.

#### 3.1.3 Fire history of study sites

All sites experienced fire at least once between 1998 and 2018, except for one Parkland and one Established Streetscape site. Two sites, both in the Native Vegetation class, were recorded as having been burnt every year. The majority of fires occurred in the mid‐dry season when conditions are optimum for fuel loads from fallen leaf litter and flammability is at a maximum.

**FIGURE 2:**
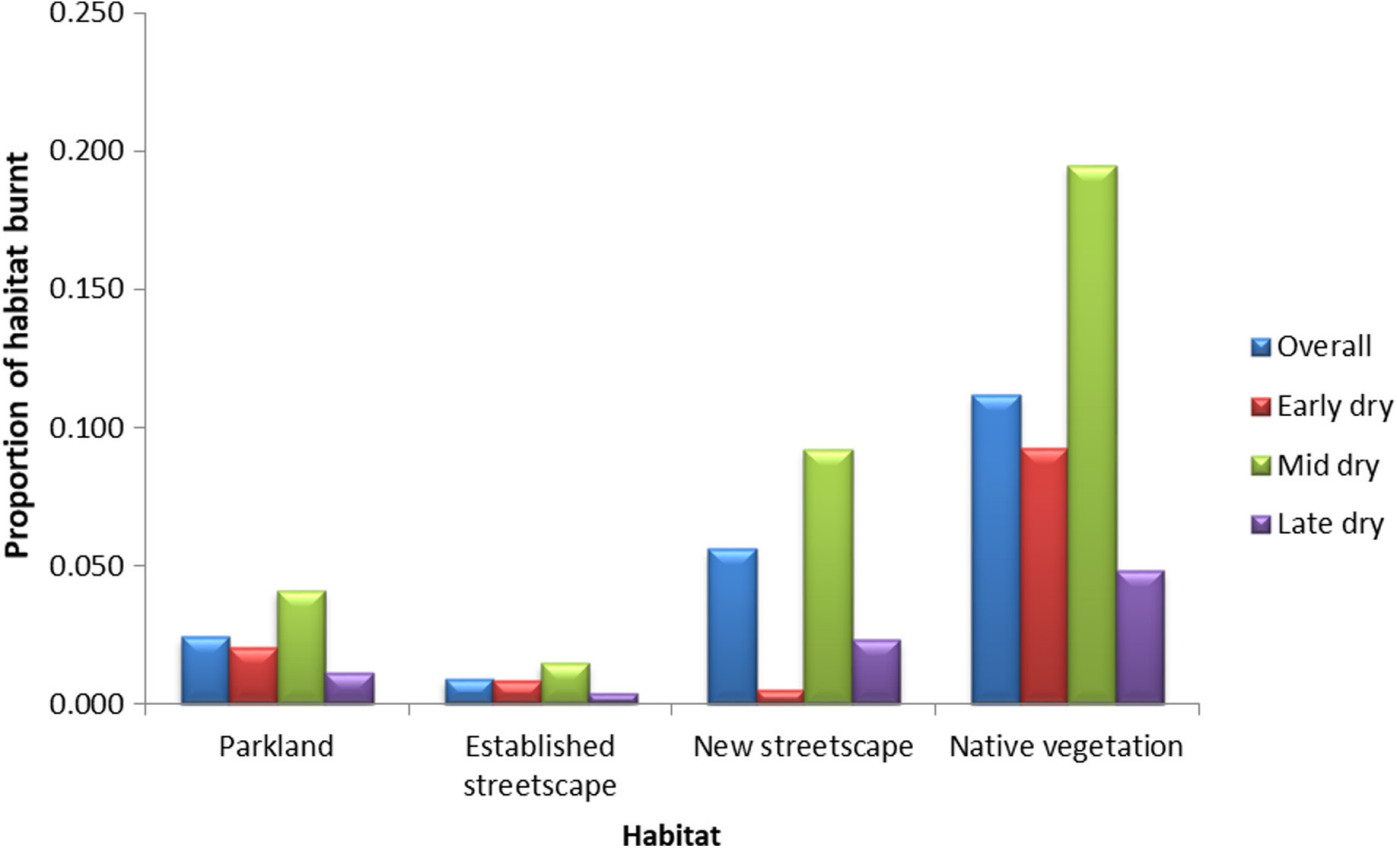
Total average proportion of habitat burnt for all sites in each habitat for all years.

Combining data for all habitat types, the highest average proportion of sites burnt was that of the Native Vegetation with very little fire occurring in areas classified as Established Streetscapes (Figure 2).

### 3.2 The effect of changing land use on fire regimes

Results of the GLMM indicated that an increase in urbanization significantly affected the fire occurrence (*p* = .038, *n* = 128), with 66.7% of sites showing a decrease, but had no statistically significant effect on the mean % burnt. The interaction between greenspace change and habitat indicated that the effects on fire occurrence were habitat dependent with an effect on fire occurrence found to be statistically significant only in Parkland habitats (*p* = .043, *n* = 128).

When we analyzed the effect of land‐use change on the timing of fire, urban change had an effect during each seasonal period on fire occurrence (Table 4). No change to the amount of urbanization showed a significant effect on the mean % burnt only in the late dry season (*p* = .025, *n* = 128). The interaction between an increase in urban change and New Streetscape habitats in the early dry season was found to be statistically significant with regard to fire occurrence, as was the interaction between an increase in urban change and Native Vegetation habitats in the early and late dry seasons. A statistically significant interaction between Parkland habitats and greenspace with respect to fire occurrence was observed in the mid dry season period.

**TABLE 4:** Results of GLMM showing the effect of land use change on the seasonality of fire (statisticallysignificant effects, *p* < 0.05, shown).

	Estimate	Std. error	*t*‐Value	*p = 005*
Early dry season				
Urban change				
No change	1.000	0.417	2.401	**.018**
Urban change × Habitat				
Increase:Native Vegetation	−1.635	0.805	−2.033	**.044**
Increase:New Streetscape	−1.891	0.829	−2.281	**.024**
Mid dry season				
Urban change				
Increase	0.720	0.323	2.231	**.028**
Greenspace change × Habitat				
No change:Parklands	3.556	1.584	2.245	**.027**
Late dry season				
Urban change				
Increase	0.864	0.321	2.692	**.008**
No change	1.220	0.410	2.974	**.004**
Urban change × Habitat				
Increase:Native Vegetation	−1.732	0.801	−2.162	**.033**

*Note*: Dependent variables: Fire occurrence and mean % burnt for each seasonal period (early, mid, and late dry season); fixed effects: urban change (increase, decrease, no change), greenspace change (increase, decrease, no change); interactions between: urban change and habitat, greenspace change, and habitat; *n* = 128.

### 3.3 The effect of land‐use change and fire regimes on bird communities

Fire regimes had a statistically significant effect on five of the eight feeding guild categories (Table 5). The proportion of frugivores, nectarivores and seed‐eating species increased in every habitat type and overall with the interaction between urban change and fire regime having a statistically significant effect on frugivores. Omnivores were found to be affected by the interaction between greenspace change and habitat. The proportion of omnivorous species increased in three out of four habitats and remained stable in one. Invertebrate, vertebrate feeders and raptors decreased overall with invertebrate feeders decreasing in every habitat; vertebrate feeders decreased in three habitats (Parklands, Established and New Streetscapes) and remained unchanged in the Native Vegetation environments; raptors decreased in the Parkland and Native Vegetation habitats.

**TABLE 5:** Results of GLMM showing the effect of land use change and fire regimes on bird assemblages in studysites (note: only statistically significant results, *p* < 0.05, shown).

	Estimate	Std. error	*t*‐Value	*p = 005*
Fruit				
Mean % burnt				
No change	0.080	0.038	2.099	**.047**
Urban change × Mean % burnt				
Increase:No change	0.163	0.078	2.101	**.050**
Invertebrates				
Mean % burnt				
No change	−0.245	0.093	−2.641	**.015**
Nectar				
Fire occurrence				
No change	0.078	0.032	2.463	**.022**
Mean % burnt				
No change	0.117	0.052	2.249	**.034**
Omnivore				
Greenspace change × Habitat				
Increase × New streetscape	0.107	0.048	2.209	**.041**
Raptor				
Fire occurrence				
No change	−0.047	0.022	−2.109	**.046**
Mean % burnt				
No change	−0.084	0.036	−2.359	**.027**
Seed				
Mean % burnt				
No change	0.171	0.055	3.129	**.005**

*Note*: Dependent variables: Changes in guild member proportion by primary feeding guild; fixed effects: land‐use change (increase, decrease, no change), fire occurrence, and mean % burnt (increase, decrease, no change); interactions between: land‐use change and habitat, land‐use change, and fire occurrence land‐use change and mean % burnt, *n* = 26.

## 4 DISCUSSION

Land‐use change and inappropriate fire regimes are two of the greatest threats to global biodiversity (Bowman et al., 2020; Driscoll et al., 2021; He et al., 2019), yet how interactions between these two stressors affect species and ecosystems is poorly understood. To our knowledge, ours is the first study to investigate the direct interactive effects of land‐use change and fire on not only avian communities but any vertebrate species and one of the very few empirical studies in this field.

Consistent with earlier studies (Allan & Willson, 1995; Price & Bradstock, 2014; Syphard et al., 2007), we found that land‐use change, specifically urbanization, significantly affected both fire occurrence and the seasonality of fire, and the majority of avian feeding guilds. The strongest interactions between urban change and mean % burnt were for those species that primarily feed on fruit where an increase in urbanization with no change to the mean % burnt was statistically significant. The Australian tropical savannas have a relatively high abundance of fruit‐bearing trees (Vigilante & Bowman, 2004), and studies investigating their response to fire have shown that fruit production peaks in areas that are unburnt (Bowman & Prior, 2004; Vigilante & Bowman, 2004). This natural bounty coupled with the fruiting plants available in urban areas, particularly in parks and established streetscapes where fires are suppressed and gardens are mature, provide a substantial source of food for frugivores. Fruits sustain not only specialist frugivores but also, during the wet season, a large proportion of nectarivorous birds (Woinarski et al., 2000). This flexibility in diet may explain our findings of an increase in nectar‐feeding species like that of primary frugivores. Additionally, a year‐round food source is effectively created by the combination of native fruiting vegetation and that found in the urban zone could feasibly increase the number of safe nesting sites for both fruit and nectar‐feeding species, allowing members of these feeding guilds greater reproductive success (Reynolds et al., 2019; Taylor et al., 2013).

Fluctuation in bird assemblages in the tropical savannas that make up the majority of the environs of the Darwin region is considered common due to the effect of seasonality upon resources (Valentine et al., 2012; Woinarski et al., 2000). It is therefore not unexpected that fire regimes had a direct effect on the majority of the feeding guilds. A statistically significant effect on species proportion was recorded for both raptors and those species that feed primarily on invertebrates when there was no change in fire regimes. In assemblages, the total proportion of raptors declined overall and in two of the four habitats; invertebrate feeders declined in every habitat and overall. In contrast, fruit, nectar, and seed‐eating species showed a positive response even though there had been no change to the fire regimes. Again, this may be attributed to these species exploiting the urban and peri‐urban landscape where fires are more controlled, and sources of food are more plentiful.

Raptors of the Darwin region, particularly Black Kites (*Milvus migrans*) and Whistling Kites (*Haliastur sphenurus*) are often seen in large numbers during and after fire events, as they prey on species escaping the fire, or feed on carrion left behind (Braithwaite & Estbergs, 1987–88; Bonta et al., 2017). That a significant negative response by raptors to no change in the fire regimes was found in this study might suggest that the fire events in the urban/peri‐urban zone are no longer optimal to sustain raptor populations. Invertebrate feeders such as Pied Butcherbirds (*Cracticus nigrogularis picatus*), Magpie‐larks (*Grallina cyanleuca neglecta*) and Black‐faced Cuckoo‐shrikes (*Coracina novaehollandiae melanops*), have also been known to exploit resources exposed at fire sites, hours or even a month later (Woinarski, 1999); given the lack of change to fire regimes, urbanization of the environment might now be responsible for lower numbers.

The decline of invertebrate‐feeding species in all habitats and overall was unexpected. Some research has been previously undertaken investigating the responses of indigenous invertebrates, specifically insects, to fire regimes or urbanization, but, as with birds, the interaction effect of these two stressors has not yet been considered. Orgeas and Anderson (2001) found that beetle numbers and diversity in an experimental study in nearby Kakadu National Park were influenced by fire occurrence but not fire timing. However, Andersen (2021) noted that fire had very little effect on the insect communities in this experiment overall. Mata et al. (2021), investigating the effect of urbanization on insects within suburban Melbourne, stressed the importance of indigenous vegetation to sustain native insect populations and noted that greenspace such as gardens or parks is often comprised of exotic plant species. Previous studies of tropical parkland areas found that insectivores were positively affected by increasing park size (Chang & Lee, 2016; Zhou & Chu, 2012). Additionally, Korányi et al. (2021) reported an increase in the proportion of birds with larger body mass when the amount of greenspace in parks increased but this was not found in our research. The formation of parklands and suburban gardens is associated with an increase in the proportion of land covered by grassy, lawned areas, and non‐native plant species. These changes to the original environment may adversely affect native invertebrate populations and their predators.

Due to the lack of similar studies, it is difficult to compare our findings to other research. Beal‐Neves et al. (2020), using time since fire as a variable, examined fire disturbance and urbanization on pollinating faunas, specifically insects, in South Brazilian grasslands and found that fire disturbance events became more frequent with an increase in urbanization; further, they found that the richness of floral visitors was positively correlated with an increase in urbanization. Conversely, Kivlin et al. (2021), investigated the response of mycorrhizal fungi to urbanization and fire in the United States and while they found that urbanization and fire affected some fungal metrics independently, no interaction between urbanization and fire was identified. However, neither of these studies involved vertebrate species and all sampling sites were within either National or State parks surrounded by urban zones. In Australia, Ramalho et al. (2018), found that the interactive effects of fire regimes and habitat loss may lead to the decline of the southern brown bandicoot; however, this was a simulative study using population viability analysis to model predictive outcomes.

One caution about our current study was that we relied on retrospective data (earlier survey data from the Bird Atlas) and, ergo, had to trust the accuracy and veracity of unknown data collectors. Subsequent research would benefit from having consecutive bird surveys undertaken by the same observer or group of surveyors. Furthermore, the quality of satellite imagery has vastly improved over time; earlier imagery lacks the clarity and resolution of later imagery. Satellite visit times have also increased in frequency allowing for more images to be available in later years, thus increasing the chances of cloud‐free, or minimal cloud, images. As technology improves, the quality of data for comparison across time will improve further. While every effort was made to confirm fire occurrence and the size of burnt areas, it must be acknowledged that smaller fires may have gone undetected in the earlier images.

Despite being able to contribute to the comparatively meager store of knowledge regarding land‐use change and fire regime interaction and their influence on vertebrate communities, our study prompts further research. Examining invertebrate‐preferring species more closely may explain why they appear sensitive to changes to their primary food source despite potentially having other sources of food; this would be greatly aided by research into the combined effects of land‐use change and fire regimes on native insect communities of the Darwin region. Consideration must be given, too, as to why vertebrate and vegetation feeders were unaffected by either land‐use change or fire regimes.

## 5 CONCLUSION

Our study aimed to investigate the effect of changing land use and associated fire regime change on the guild structures of the Darwin avian assemblages. While land‐use change is causing little change to the avian assemblage, we determined that altered land use on fire regimes is having an indirect influence on urban bird community structures. We have also demonstrated the interactive effects of land‐use change, particularly an increase in urbanization, and fire regimes can impact specific feeding guilds. Overall, a deeper analysis of specific species within feeding guilds and individual species reactions to land‐use change and fire regimes is necessary to be able to best formulate conservation strategies and management plans for ecosystems within the urban environment.

## AUTHOR CONTRIBUTIONS

**Sarah Fischer:** Conceptualization (lead); data curation (lead); formal analysis (lead); investigation (lead); methodology (lead); writing – original draft (lead); writing – review and editing (equal). **Andrew C. Edwards:** Conceptualization (supporting); methodology (supporting); writing – original draft (supporting); writing – review and editing (equal). **Stephen T. Garnett:** Methodology (supporting); writing – original draft (supporting); writing – review and editing (equal). **Timothy G. Whiteside:** Methodology (supporting); writing – original draft (supporting); writing – review and editing (equal). **Patrice Weber:** Methodology (supporting); writing – original draft (supporting); writing – review and editing (supporting).

## CONFLICT OF INTEREST STATEMENT

The authors declare no competing interests.

## Data Availability

Data used in analysis will be made publicly available via the Charles Darwin University Research Webportal https://researchers.cdu.edu.au/en/.

## Appendix

**Table A1:** Total species recorded.

Order	Species	Survey years recorded	
		1999/2000	2019/2020
Accipitriformes	Black Kite (*Milvus migrans*)	√	√
	Black‐shouldered Kite (*Elanus axillaris*)	√	X
	Brahminy Kite (*Haliastur indus*)	√	√
	Brown Goshawk (*Accipiter fasciatus didimus*)	√	√
	Collared Sparrowhawk (*Accipiter cirrocephalus*)	X	√
	Little Eagle (*Hieraaetus morphnoides*)	√	X
	Osprey (*Pandion cristatus*)	√	X
	Pacific Baza (*Aviceda subcristata*)	√	X
	Square‐tailed Kite (*Lophoictinia isura*)	X	√
	Wedge‐tailed Eagle (*Aquila audax fleayi*)	√	X
	Whistling Kite (*Haliastur sphenurus*)	√	√
	White‐bellied Sea‐Eagle (*Haliaeetus leucogaster*)	√	X
Anseriformes	Magpie Goose (*Anseranas semipalmata*)	√	√
	Radjah Shelduck (*Tadorna radjah*)	X	√
Caprimulgiformes	Tawny Frogmouth (*Podargus strigoides phalaenoides*)	√	X
Charadriiformes	Australian Pratincole (*Stiltia isabella*)	√	X
	Bar‐tailed Godwit (*Limosa lapponica*)	√	X
	Beach Stone‐curlew (*Esacus neglectus*)	√	X
	Black‐fronted Dotterel (*Elseyornis melanops*)	√	X
	Black‐winged Stilt (*Himantopus himantopus*)	√	X
	Bush Stone‐curlew (*Burhinus grallarius*)	√	√
	Common Greenshank (*Tringa nebularia*)	√	X
	Common Sandpiper (*Actitis hypoleucos*)	√	X
	Curlew Sandpiper (*Calidris ferruginea*)	√	X
	Eastern Curlew (*Numenius madagascariensis*)	√	X
	Great Knot (*Calidris tenuirostris*)	√	X
	Greater Sand Plover (*Charadrius* leschenaultii)	√	X
	Gray Plover (*Pluvialis squatarola*)	√	X
	Gray‐tailed Tattler (*Tringa brevipes*)	√	X
	Lesser Sand Plover (*Charadrius mongolus*)	√	X
	Little Curlew (*Numenius minutus*)	√	X
	Marsh Sandpiper (*Tringa stagnatilis*)	√	X
	Masked Lapwing (*Vanellus miles miles*)	√	√
	Pacific Golden Plover (*Pluvialis fulva*)	√	X
	Red Knot (*Calidris canutus*)	√	X
	Red‐backed Button‐quail (*Turnix maculosus*)	√	X
	Red‐capped Plover (*Charadrius ruficapillus*)	√	X
	Red‐necked Stint (*Calidris ruficollis*)	√	X
	Ruddy Turnstone (*Arenaria interpres*)	√	X
	Sanderling (*Calidris alba*)	√	X
	Sharp‐tailed Sandpiper (*Calidris acuminata*)	√	X
	Sooty Oystercatcher (*Haematopus fuliginosus*)	√	X
	Swinhoe's Snipe (*Gallinago megala*)	√	X
	Terek Sandpiper (*Xenus cinereus*)	√	X
	Whimbrel (*Numenius phaeopus*)	√	X
	Wood Sandpiper (*Tringa glareola*)	√	X
Columbiformes	Bar‐shouldered Dove (*Geopelia humeralis*)	√	√
	Brown‐capped Emerald‐Dove (*Chalcophaps longirostris*)	√	X
	Crested Pigeon (*Ocyphaps lophotes*)	√	X
	Peaceful Dove (*Geopelia striata placida*)	√	√
	Rock Dove (*Columba livia*)	√	X
	Rose‐crowned Fruit‐Dove (*Ptilinopus regina*)	√	X
	Torresian Imperial‐Pigeon (*Ducula bicolor*)	√	√
Coraciiformes	Azure Kingfisher (*Ceyx azureus ruficollaris*)	√	√
	Blue‐winged Kookaburra (*Dacelo leachii leachii*)	√	√
	Collared Kingfisher (*Todiramphus chloris sordidus*)	√	X
	Dollarbird (*Eurystomus orientalis*)	√	√
	Forest Kingfisher (Todiramphus *macleayii macleayii*)	√	√
	Little Kingfisher (*Ceyx pusilla ramsayi*)	X	√
	Rainbow Bee‐eater (*Merops ornatus*)	√	√
	Red‐backed Kingfisher (*Todiramphus pyrrhopygius*)	√	X
	Sacred Kingfisher (*Todiramphus sanctus*)	√	X
Cuculiformes	Brush Cuckoo (*Cacomantis variolosus*)	√	√
	Channel‐billed Cuckoo (*Scythrops novaehollandiae*)	√	√
	Eastern Koel (*Eudynamys orientalis subcyanocephala*)	√	√
	Horsfield's Bronze Cuckoo (*Chalcites basalis*)	√	X
	Little Bronze Cuckoo (*Chalcites minutillus minutillus*)	√	√
	Oriental Cuckoo (*Cuculus optatus*)	√	X
	Pheasant Coucal (*Centropus phasianinus melanurus*)	√	√
Falconiformes	Australian Hobby (*Falco longipennis*)	√	X
	Black Falcon (*Falco subniger*)	√	X
	Brown Falcon (*Falco berigora*)	√	X
	Nankeen Kestrel (*Falco cenchroides*)	√	X
	Peregrine Falcon (*Falco peregrinus*)	√	X
Galliformes	Brown Quail (*Coturnix ypsilophora australis*)	√	X
	King Quail (*Synoicus chinensis*)	√	X
	Orange‐footed Scrubfowl (*Megapodius reinwardt*)	√	√
Gruiformes	Brolga (Grus *rubicunda*)	√	X
	Buff‐banded Rail (*Hypotaenidia philippensis*)	√	X
	Chestnut Rail (*Eulabeornis castaneoventris*)	√	X
	Pale‐vented Bush‐hen (*Amaurornis moluccana*)	√	X
Passeriformes	Australasian Figbird (*Sphecotheres vieilloti ashbyi*)	√	√
	Australasian Pipit (*Anthus novaeseelandiae*)	√	X
	Banded Honeyeater (*Cissomela pectoralis*)	X	√
	Bar‐breasted Honeyeater (*Ramsayornis fasciatus*)	√	√
	Black Butcherbird (*Cracticus quoyi*)	√	√
	Black‐faced Cuckoo‐shrike (*Coracina novaehollandiae melanops*)	√	√
	Black‐faced Woodswallow (*Artamus cinereus melanops*)	√	√
	Blue‐faced Honeyeater (*Entomyzon cyanotis albipennis*)	√	√
	Broad‐billed Flycatcher (*Myiagra ruficollis*)	√	X
	Brown Honeyeater (*Lichmera indistincta indistincta*)	√	√
	Chestnut‐breasted Mannikin (*Lonchura castaneothorax*)	√	√
	Cicadabird (*Edolisoma tenuirostris*)	√	X
	Crimson Finch (*Neochmia phaeton phaeton*)	√	√
	Double‐barred Finch (*Taeniopygia bichenovii* annulosa)	√	√
	Dusky Honeyeater (*Myzomela obscura obscura*)	√	√
	Fairy Martin (*Petrochelidon ariel*)	√	X
	Golden‐headed Cisticola (*Cisticola exilis*)	√	X
	Great Bowerbird (*Ptilonorhynchus nuchalis* nuchalis)	√	√
	Green‐backed Gerygone (*Gerygone chloronota*)	√	√
	Gray Butcherbird (Cracticus *torquatus colletti*)	√	√
	Gray Fantail (*Rhipidura albiscapa*)	√	X
	Gray Shrike‐thrush (*Colluricincla harmonica*)	√	X
	Gray Whistler (*Pachycephala simplex simplex*)	√	√
	Gray‐crowned Babbler (*Pomatostomus temporalis rubeculus*)	√	√
	Helmeted Friarbird (*Philemon buceroides ammitophila*)	√	√
	Horsfield's Bushlark (*Mirafra javanica*)	√	X
	Jacky Winter (*Microeca fascinans pallida*)	X	√
	Large‐billed Gerygone (*Gerygone magnirostris magnirostris*)	√	√
	Leaden Flycatcher (*Myiagra rubecula concinna*)	√	X
	Lemon‐bellied Flycatcher (*Microeca flavigaster flavigaster*)	√	√
	Little Friarbird (*Philemon citreogularis sodidus*)	√	√
	Little Shrike‐thrush (*Colluricincla megarhyncha parvula*)	√	X
	Little Woodswallow (*Artamus minor*)	√	X
	Long‐tailed Finch (*Poephila acuticauda hecki*)	√	√
	Magpie‐lark (*Grallina cyanoleuca neglecta*)	√	√
	Mangrove Gerygone (*Gerygone levigaster levigaster*)	√	√
	Mangrove Golden Whistler (*Pachycephala melanura melanura*)	√	√
	Mangrove Gray *Fantail* (*Rhipidura phasiana*)	X	√
	Mangrove Robin (*Peneothello pulverulenta*)	√	X
	Masked Finch (*Poephila personata personata*)	√	X
	Mistletoebird (*Dicaeum hirundinaceum*)	√	√
	Northern Fantail (*Rhipidura rufiventris*)	√	√
	Northern Restless *Flycatcher* (*Myiagra inquieta nana*)	X	√
	Olive‐backed Oriole (*Oriolus sagittatus affinis*)	√	√
	Paperbark Flycatcher (*Myiagra nana*)	X	√
	Pied Butcherbird (*Cracticus nigrogularis picatus*)	√	√
	Rainbow Pitta (*Pitta iris iris*)	√	X
	Red‐backed Fairy‐wren (*Malurus melanocephalus cruentatus*)	√	√
	Red‐headed Honeyeater (*Myzomela erythrocephala erythrocephala*)	√	√
	Restless Flycatcher (*Myiagra inquieta nana*)	√	X
	Rufous Fantail (*Rhipidura rufifrons*)	√	X
	Rufous Whistler (*Pachycephala rufiventris falcata*)	√	√
	Rufous‐banded Honeyeater (*Conopophila albogularis*)	√	√
	Rufous‐throated Honeyeater (*Conopophila rufogularis*)	√	√
	Shining Flycatcher (*Myiagra alecto melvillensis*)	√	X
	Silver‐crowned Friarbird (*Philemon argenticeps argenticeps*)	√	√
	Spangled Drongo (*Dicrurus bracteatus baileyi*)	√	√
	Striated Pardalote (*Pardalotus striatus uropygialis*)	√	√
	Torresian Crow (*Corvus orru*)	√	√
	Tree Martin (*Petrochelidon nigricans*)	√	√
	Varied Sittela (*Daphoenositta chrysoptera*)	X	√
	Varied Triller (*Lalage leucomela rufiventris*)	√	√
	Weebill (*Smicrornis brevirostris flavescens*)	√	√
	White‐bellied Cuckoo‐shrike (*Coracina papuensis hypoleuca*)	√	√
	White‐breasted Whistler (*Pachycephala lanioides*)	√	X
	White‐breasted Woodswallow (*Artamus leucorynchus*)	√	X
	White‐gaped Honeyeater (*Stomiopera unicolor*)	√	√
	White‐throated Honeyeater (*Melithreptus albogularis*)	√	√
	White‐winged Triller (*Lalage sueurii*)	√	√
	Willie Wagtail (*Rhipidura leucophrys picata*)	√	X
	Yellow Oriole (*Oriolus flavocinctus* flavocinctus)	√	√
	Yellow Wagtail (*Motacilla flava tschutschensis*)	√	X
	Yellow White eye (*Zosterops luteus luteus*)	√	√
	Yellow‐throated Miner (*Manorina flavigula lutea*)	√	√
	Zitting Cisticola (*Cisticola juncidis leanyeri*)	√	X
Pelecaniformes	Australian White Ibis (*Threskiornis moluccus*)	√	√
	Black Bittern (*Ixobrychus flavicollis*)	√	X
	Black‐necked Stork (*Ephippiorhynchus asiaticus*)	√	X
	Cattle Egret (*Ardea ibis*)	√	X
	Eastern Reef Egret (*Egretta sacra*)	√	X
	Glossy Ibis (*Plegadis falcinellus*)	√	X
	Great Egret (*Ardea modesta*)	√	X
	Great‐billed Heron (*Ardea sumatrana*)	√	X
	Intermediate Egret (*Ardea intermedia*)	√	√
	Little Egret (*Egretta garzetta*)	√	X
	Nankeen Night‐Heron (*Nycticorax caledonicus*)	√	X
	Pied Heron (*Egretta picata*)	√	√
	Royal Spoonbill (*Platalea regia*)	√	X
	Straw‐necked Ibis (*Threskiornis spinicollis*)	√	√
	Striated Heron (*Butorides striatus stagnatilis*)	√	X
	White‐faced Heron (*Egretta novaehollandiae*)	√	X
Psittaciformes	Galah (Eolophus *roseicapillus*)	√	√
	Little Corella (*Cacatua sanguinea sanguinea*)	√	√
	Northern Rosella (*Platycercus venustus*)	√	X
	Red‐collared Lorikeet (*Trichoglossus haematodus rubritorquis*)	√	√
	Red‐tailed Black Cockatoo (*Calyptorhynchus banksii macrorhychus*)	√	√
	Red‐winged Parrot (*Aprosmictus erythropterus coccineopterus*)	√	√
	Sulfur‐crested Cockatoo (*Cacatua galerita fitzroyi*)	√	√
	Varied Lorikeet (*Psitteuteles versicolor*)	√	X
